# The genetic architecture of temperature-induced partial fertility restoration in *A*_1_ cytoplasm in sorghum (*Sorghum bicolor* (L.) Moench)

**DOI:** 10.1007/s00122-025-04946-4

**Published:** 2025-07-02

**Authors:** D. R. Jordan, R. R. Klein, J. Melonek, I. Small, A. Cruickshank, L. Bradburn, S. Malory, Y. Tao, A. Hathorn, C. H. Hunt, L. T. Amenu, E. S. Mace

**Affiliations:** 1 Queensland Alliance for Agriculture and Food Innovation, The University of Queensland, Warwick, QLD 4370 Australia; 2 Southern Plains Agricultural Research Center, USDA-ARS, College Station, TX 77845 USA; 3 School of Molecular Sciences, The University of Western Australia, Perth, WA 6009 Australia; 4 Hermitage Research Facility, Department of Primary Industries, Warwick, QLD 4370 Australia; 5 Department of Agriculture and Fisheries, Gatton Research Station, Gatton, QLD 4343 Australia; 6 Agricultural Genomics Institute, Shenzhen, China; 7 Queensland Alliance for Agriculture and Food Innovation, The University of Queensland, Brisbane, QLD 4370 Australia

## Abstract

**Key message:**

High-temperature-induced partial fertility in CMS sorghum is controlled by multiple genes that are distinct from genes involved in fertility restoration, contributing to reduced diversity in elite females.

**Abstract:**

Cytoplasmic male sterility (CMS) is used for commercial production of hybrid seed in sorghum. CMS-based hybrid breeding systems require female parental lines (CMS lines) to remain male sterile to prevent self-pollination and enable cross-pollination to generate hybrid seed. However, genetic and environmental factors can lead to the loss of male sterility in the pollen-accepting female parent, resulting in the production of contaminating non-hybrid seeds through self-fertilization with large economic consequences. It is known that high temperatures around flowering time induce sterility breakdown, or partial fertility; however, the genetic control of this phenomenon is poorly understood. To investigate the molecular processes controlling sterility breakdown, a large association mapping population of elite CMS parental lines was used to map the genomic regions controlling partial fertility. In this study, we used genome-wide association studies on a panel of 2049 sorghum lines grown in six field trials at Emerald Queensland representing six different environments. The seed planting was set up in such a way that flowering corresponded with the hottest part of the year. In total 43 significant SNPs were identified, indicating that the trait is controlled by multiple genes; however, previously identified major genes for fertility restoration were not the main cause of partial fertility. Diversity and linkage disequilibrium decay patterns in separate elite male and CMS pools also indicated the constraints on genetic diversity within the female parents due to partial fertility, rather than the frequency of the previously identified major fertility restoration genes. The understanding of the control of sterility breakdown provides new avenues for trait introgression in elite female pools.

**Supplementary Information:**

The online version contains supplementary material available at 10.1007/s00122-025-04946-4.

## Introduction

Cytoplasmic male sterility (CMS) systems are used for commercial production of hybrid seed in a range of crops including maize, sunflowers, canola and sorghum (Bohra et al. 2016). CMS was first reported in sorghum by Stephens and Holland (1954) with the first commercial sorghum hybrids being grown in the early 1960s. In sorghum, CMS is operationalized using a three-line system often described using the three-letter coding system *A*/*B* and *R* where different combinations of nuclear and cytoplasmic genes result in sterility or fertility (Kim and Zhang 2018). Cost-effective production of *F*_1_ seed is achieved by growing male sterile “*A*” lines or female parents unable to produce functional pollen in isolated crossing blocks with male fertile “*R*” lines. Sterility in “*A*” lines is the result of mitochondrial genes in the maternally inherited cytoplasm that prevent the formation of viable pollen (CMS) (Hanson and Bentolila 2004). New female parents are developed as “*B*” lines which have a cytoplasm that does not contain the sterility-inducing mitochondrial genes. These *B* lines are then converted to male sterile “*A*” line versions via backcrossing to a source of sterile cytoplasm. The *A*/*B* pairs then share the same nuclear genome but have different cytoplasms. The restorer or “*R*” lines are used as pollen parents in hybrid production blocks and carry dominant nuclear restorer genes that counteract the impacts of the mitochondrial genes in the “*A*” line cytoplasm allowing production of male fertile *F*_1_ hybrids (Kim and Zhang 2018).

At the molecular level, it is thought that CMS in plants involves the expression of mitochondrial genes that produce gene products that interfere with normal pollen production (Chase 2007, Fishman and Sweigart 2018; Kim and Zhang 2018). Unfortunately, the identification of these sterility-inducing genes and their mechanisms has proven to be challenging due to the complexity of mitochondrial genome organization (Kim and Zhang 2018; Bohra et al. 2016; Kazama et al. 2019). However, in recent years advances in sequencing techniques enabled identification of multiple CMS-causing genes in more than a dozen crop species including rice, *Brassicas*, maize and wheat (Kim and Zhang 2018, Melonek et al. 2019). The majority of these genes show a chimeric structure made of conserved and new DNA sequences formed through multiple rearrangements of mitochondrial sequences, as well as via substoichiometric shifting and sequence changes (Hanson and Bentolila 2004; Kim and Zhang 2018). In rice, the mitochondrial gene *WA352* has been shown to produce a protein that inhibits the nuclear-encoded mitochondrial protein COX11, thus modifying peroxide metabolism in the tapetum triggering premature tapetal programmed cell death and consequent pollen abortion (Luo et al. 2013). In contrast, the identification of the nuclear genes responsible for fertility restoration has proven to be relatively straightforward, with *Restorer-of-fertility* (*Rf*) genes being identified in a range of species including maize (Cui et al. 1996), rice (Ahmadikhah and Karlov 2006; Itabashi et al. 2011; Komori et al. 2004), sorghum (Jordan et al. 2010, 2011; Praveen et al. 2015) and wheat (Melonek et al. 2019). The cloned *Rf* genes belong overwhelmingly to the pentatricopeptide repeat (PPR) gene family (Praveen et al. 2018; Kim and Zhang 2018). PPR genes are a large family of RNA-binding proteins which regulate several aspects of gene expression in organelles including splicing, editing, RNA stabilization and cleavage (Barkan and Small 2014). The subset of the PPR genes involved in fertility restoration in crops, referred to as *Rf-like*, are usually located in genomic clusters and show high similarity to each other (Gaborieau et al. 2016; Geddy and Brown 2007). Pangenome-level analyses of clusters of the *Rf-like* clade show extreme variation in structure and gene content within and across species (Melonek et al. 2016, Melonek et al. 2019, Walkowiak et al. 2020).

In sorghum, fertility restoration is controlled by a relatively small number of major restorer genes including *Rf1* (Klein et al. 2001), *Rf2* (Jordan et al. 2010; Madugula et al. 2018), *Rf5* (Jordan et al. 2011; Kiyosawa et al. 2022), and *Rf6* (Praveen et al. 2015). In addition to genetic factors, fertility in cytoplasmic male sterility systems in sorghum was reported to be sensitive to environmental variables such as temperature (Brooking 1976) and photoperiod (Batch and Morgan 1974).

Although it has been rarely reported in the literature, partial fertility is widely observed in sorghum crosses particularly in crosses between fertile and sterile parent lines (Jordan et al. 2011). This partial fertility appears to be the result of minor effect genes such as those identified by Jordan et al. (2011), Maunder and Pickett (1959) and Miller and Pickett (1964) which give rise to a continuum of full and partial fertility observed in the progeny of *F*_1_ hybrids (Jordan et al. 2011). Partial fertility in sorghum is temperature sensitive with expression of fertility increasing when high temperatures occur around flowering (unpublished data).

In applied hybrid sorghum breeding programmes, genes for partial fertility must be excluded from female parent lines because the production of pollen by female parents leads to self-pollination and the presence of inbred lines in hybrid seed, rendering it unsaleable. In practice, this can be difficult to achieve because the partial fertility phenotype can only be observed when the nuclear genome of new female parents is backcrossed into sterile cytoplasm (Jordan et al. 2010). The situation is further complicated because expression of partial fertility increases as the proportion of recurrent parent genome increases during backcrossing (unpublished observation), requiring substantial investment in the cytoplasmic conversion of lines before the breeder can be confident that they are not likely to become partially fertile. A final complication results from the fact that the expression of partial fertility varies with environmental conditions and is only expressed when high temperatures occur at critical times around flowering. As a result, lines carrying genes for partial fertility may not be detected if the lines are not exposed to the relevant conditions at the critical development phase (Jordan et al. 2010). Little is known about the genetic control of partial fertility and to date only a single QTL for partial fertility has been mapped (Jordan et al. 2011). However, the nature of the trait, particularly its interaction with the environment and its dosage dependence, suggests a more complex inheritance compared with the major restorer genes. One practical implication of this complex genetic control is reluctance on the part of breeders to make genetically diverse crosses when developing new parental lines due to the frequency of partial fertility in these crosses (Jordan et al. 2010).

In this study we used association mapping on a large panel of CMS parent lines to identify QTL associated with the genetic control of partial fertility in sorghum and investigate its impact on hybrid breeding.

## Materials and methods

### Genetic material

#### Germplasm set 1 diverse *A* lines for association mapping

A total of 2049 female parent lines in *A*_1_ cytoplasm were grown in a trial comprising 59 lines from the Nuseed breeding programme and 1990 lines from the UQ/DAF/GRDC pre-breeding programme. The Nuseed lines consisted of a sample of germplasm from the commercial breeding programme known to vary in their expression of partial fertility. The material from the UQ/DAF/GRDC breeding programme consisted of a sample of active and historical hybrid parent lines as well as a set of new parent lines that were being developed by the programme. The UQ/DAF/GRDC parent lines shared some degree of co-ancestry.

#### Germplasm set 2 elite *A*/*B* and *R* lines for diversity analysis

Germplasm set 2 consisted of 2219 advanced *A*/*B* lines and 2135 *R* lines from the UQ/DAF/GRDC sorghum breeding programme that were active in final stage testing during the last 5 years. These lines were the result of multiple cycles of crossing and selection for performance in hybrid combination since hybrid breeding commenced in the mid-1960s. During this time, the programme has been managed in a way that is analogous to the early parental development phase of medium size commercial breeding programme. All the *A*/*B* lines in this set were either also in set 1 or were first order relatives of the lines in set 1. The *R* lines (male parents) in set 2 have been strongly selected for capacity to produce fully fertile *F*_1_ hybrids in combination with male sterile female parents in *A*_1_ cytoplasm, whereas the *B* lines (female parents) have been selected to exhibit acceptable levels of male sterility in *A*_1_ cytoplasm. Investment in female and male populations as measured by number of crosses made for each pool has been approximately equivalent during the last 30 years (unpublished data).

#### Field trial design

A total of six field trials were planted at Emerald Research Station in Queensland (Lat. −23.528767, Long. 148.212717) over 4 years from 2013 to 2016 (Table 1). The trials were planted from early October to late December to ensure that flowering would occur during the hottest part of the year. For the years in question, average maximum and minimum temperatures around the flowering period (November–February) were 35.4 °C and 21 °C, respectively, with the hottest days in these 4 months in the different years varying between a low of 37.2 °C to a high of 44.4 °C. Standard agronomic management and weed control for sorghum was used and irrigation applied when required by the crop to avoid water stress. Each plot consisted of a single 5 m row planted at 1 m row spacing. All trials used a partially replicated design (Cullis et al. 2006) with test entries replicated ~ 1.5 times and check entries replicated between 2 and 10 times. The number of entries in the trials varied between 593 and 803 genotypes and the number of plots varied between 960 and 1288 (Table 1). The concurrence of genotypes across the six trials and 4 years was sufficient for the trials to be analysed as a single multi-environment experiment (ESM Table S1). A customized row column design was used to minimize spatial error effects within each trial.

**Table 1:** Planting dates and composition of trials used in the study

Trial name	Planting date	Number of genotypes	Plots	Observations	Measured days*
Emer1403	3/10/2013	593	960	1903	4
Emer1404	18/10/2013	603	960	2809	5
Emer1505	18/11/2014	780	1200	3244	6
Emer1506	1/12/2014	783	1200	2306	6
Emer1607	23/12/2015	803	1288	5422	10
Emer1704	23/11/2016	682	1000	4311	8

*Indicates the total number of days the partial fertility trait was measured in each trial

#### Phenotyping

Sorghum heads flower in segments commencing at the top of the panicle and progressing downwards over a period of approximately 5 days. Freshly flowered anthers in sorghum only dehisce (release pollen) on the day they emerge and can be distinguished from the anthers that dehisced on the previous day by their colour. On each day of data collection, the freshly flowered portion of any heads flowering in a plot were visually rated using a 1–9 rating previously described by Jordan et al. (2011). This scale is based on the size, colour and morphology of the fresh anthers (see detailed description below) and was shown to be strongly correlated with pollen production and seed set (Jordan et al. 2011). Visible pollen and seed production commenced when anthers had a minimum rating of 6. Unpublished data had previously shown that lines with ratings of 4 or 5 under normal temperature conditions typically produce pollen when exposed to high temperatures at flowering time. On any day only a proportion of genotypes were flowering; however, the overlap of flowering periods of the genotypes enabled data from different days and trials to be analysed as a single multi-environment trial. In total, data were recorded on a combined total of 36 separate days over the six trials with between 1903 and 5422 plot × day observations being recorded for each trial giving a total of 19,995 plot × day observations across all trials. This meant that each plot in a trial was scored between 2 and 4 times on average.

#### Scoring system for anther sterility

A visual scoring system for sterility was previously developed by Jordan et al. (2011) and consisted of nine ratings. Score 1 = sterile very small colourless anthers; dehiscence pore absent; Score 2 = sterile small colourless anthers; dehiscence pore absent; Score 3 = sterile small but slightly larger again anthers, may be more coloured; dehiscence pore absent; Score 4 = sterile small, but slightly larger again, more coloured anthers; dehiscence pore absent; Score 5 = sterile medium anthers, coloured; dehiscence pore absent; Score 6 = partially fertile larger coloured anthers; dehiscence pore present in some anthers, pollen present in very small quantities; Score 7 = partially fertile, larger coloured anthers; dehiscence present in most anthers, some visible pollen; Score 8 = moderately plump coloured anthers; dehiscence pore present in all anthers and pollen present; and Score 9 = plump coloured anthers; dehiscence pore present copious amounts of pollen.

#### Genotyping and imputation

DNA was extracted from bulked young leaves of five plants of each genotype in the two populations using a previously described CTAB method (Doyle and Doyle 1987). The two germplasm sets were genotyped using medium genome-wide SNPs provided by Diversity Arrays Technology Pty Ltd (www.diversityarrays.com), which involves complexity reduction of the genomic DNA to remove repetitive sequences using methylation sensitive restriction enzymes prior to sequencing on Next Generation sequencing platforms. The sequence data generated were then aligned to version 3.1.1 of the sorghum reference genome sequence (McCormick et al. 2018) downloaded from the Phytozome 13 website (https://phytozome-next.jgi.doe.gov/) to identify single nucleotide polymorphisms (SNPs).

In total, 21,886 polymorphic SNPs were identified on the sets of lines. The overall proportion of missing data reported in the raw genotypic data sets was approximately 13%. Individual SNP markers with > 40% missing data were removed from further analysis and the remaining missing values were imputed using Beagle v5.0 (Browning and Browning 2016; Browning et al. 2018). An average imputation accuracy of 96% was achieved across both populations.

### Statistical analysis

Data from multiple trials measured over multiple days were combined into a Multi-Environment Trial (MET). The fertility scores were analysed using a linear mixed model which contained both fixed and random effects. Fixed effects included a mean value for each trial and a mean value for each date within each trial. Trials were assessed for possible spatial field effects, and spatial terms were included for each trial, as necessary. These random spatial effects included auto-regression correlations for rows and columns for each trial correlated across dates.

Correlations between dates and trials were examined by fitting genotype effects as random with correlation between each trial/date combination. These results showed very high genetic correlations between trials and dates which allowed the inclusion of a fixed genotype main effect. By fixing all the non-genetic random effects in the model, Best Linear Unbiased Estimates (BLUEs) were calculated that were representative of genotype performance across all trials and all dates. These BLUEs were used in the subsequent genome-wide association study (GWAS). All statistical analyses were conducted with the *R* software (www.R-project.org) using the package ASReml-R version 3 (Butler et al. 2009).

### Association mapping

The imputed SNP data set for germplasm set 1 was further filtered on MAF > 0.001, resulting in a final set of 18,638 SNPs used for GWAS. The GWAS analysis was conducted using BLINK (Huang et al. 2019), in which population structure was controlled using the first three principal components generated from a principal component analysis of SNP data. The GEC software package (Li et al. 2012) was used to calculate the number of independent tests within the SNP data. Based on this number, the Bonferroni correction was applied to set significant threshold for the association analysis at 5.78E−6.

### Diversity analysis of *B* and *R* line pools

A principal component analysis was conducted on germplasm set 2 which consisted of elite female (B) and male (R) lines from the UQ/DAF/GRDC pre-breeding programme. Nucleotide diversity was estimated for each SNP by calculating the average number of pairwise differences between sequences (*π*). Average *π* values across linkage groups and the whole genome were computed for the *B* and *R* groups using vcftools (Danecek et al. 2011). Linkage disequilibrium (LD) was estimated for each pair of SNPs by calculating the correlation coefficient, $r^{2}$. The $r^{2}$ values for the *B* and *R* groups were computed using PopLDdecay (Zhang et al. 2019), and their LD decay curves were compared with those of the Australian sorghum diversity panel (Tao et al. 2020).

### Candidate genes

The location of SNPs significantly associated with partial fertility was compared with the genomic locations of four previously cloned fertility restorer genes, *Rf1*, *Rf2*, *Rf5* and *Rf6* (Jordan et al. 2010, 2011; Klein et al. 2001; Praveen et al. 2015; Madugula et al. 2018; Kiyosawa et al. 2022), and against a set of 63 candidate genes identified by Dhaka et al. (2020) that were selected as strong candidates for engineering male fertility in sorghum based on variable expression during anther development in sorghum and/or their rice orthologs have been experimentally shown to play role in anther development.

## Results

**Fig. 1:**
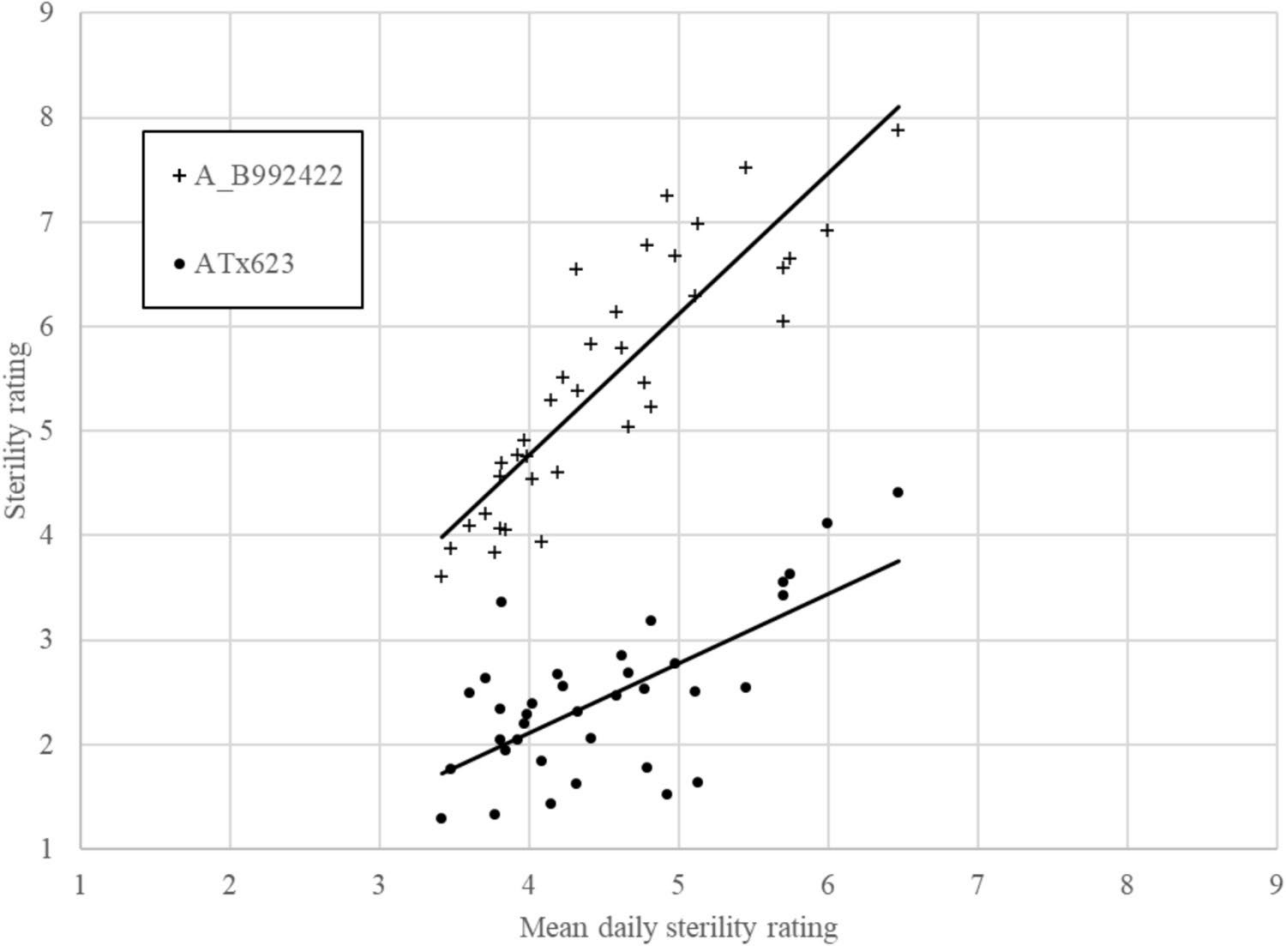
BLUEs of daily sterility ratings for two CMS lines displaying contrasting responses to high temperatures during flowering plotted against estimates of daily sterility rating for the entire dataset. ATx623 is a highly sterile female that does not become fertile under high temperatures, and A_B992422 is known to become fertile in response to high temperatures around flowering

Average pairwise correlations of sterility scores for the same genotypes scored on different days were high, varying from 0.62 to 0.99 with an average of 0.78 indicating relatively low levels of crossover GxE interactions. This enabled the data from the six experiments and 36 days to be analysed jointly to produce a single best linear unbiased estimate (BLUE) for each line used for GWAS. The repeatability of the across site estimate was high at 0.8 indicating that genetic effects explain most of the variation in the trait in these experiments and that crossover GxE was limited. This is further supported by Fig. 1 which shows an estimate of sterility rating for each day for two “*A*” lines (ATx623 and A_B992422), which were used as checks across all the experiments, plotted against the estimate of the average sterility rating for a particular day for all genotypes plotted in order of ascending value. These two lines are phenotypic extremes. ATx623 is a highly sterile genotype which does not break down whereas A_B992422 is known to become fertile in response to high temperatures around flowering. Both lines show relatively similar linear positive responses in sterility rating as the overall sterility rating of the day increases indicating that crossover GxE interaction is limited.

**Fig. 2:**
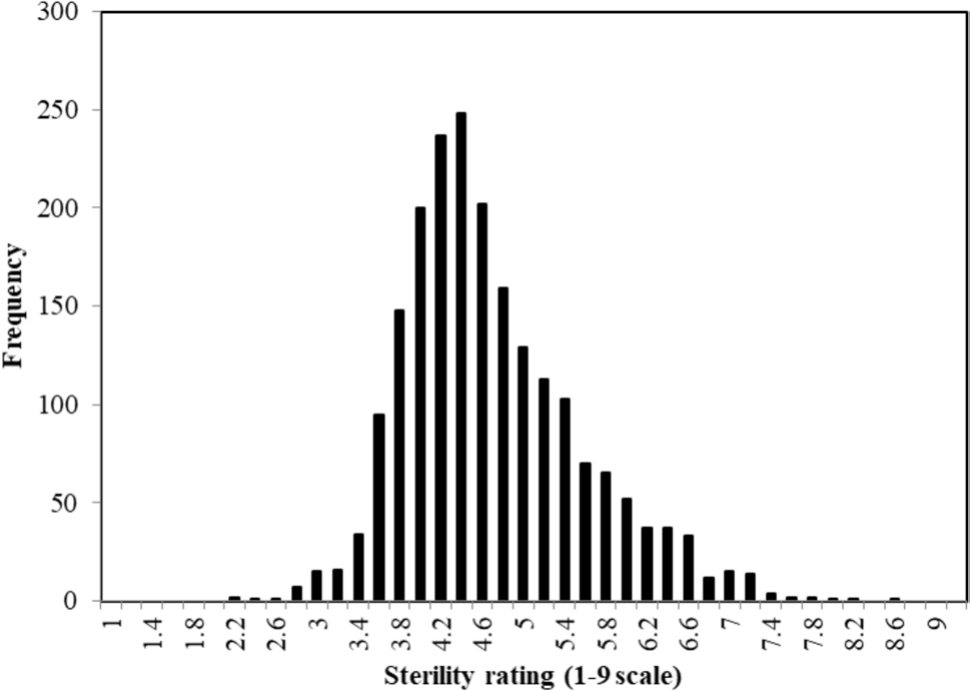
Histogram of phenotypic values of overall sterility ratings for 2049 CMS lines

A histogram showing the distribution of the overall BLUEs of sterility ratings for the 2049 CMS lines is presented in Fig. 2. The distribution of phenotypic values was relatively normally distributed and ranged from 2.2 to 8.6 with an average of 4.6.

### Association mapping

**Fig. 3:**
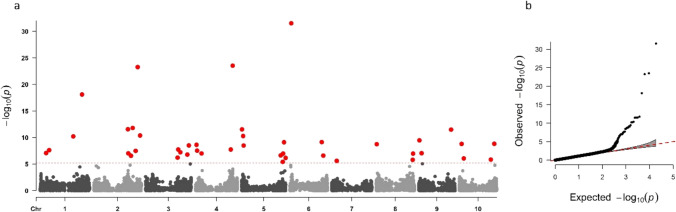
GWAS of sterility ratings, with significant markers above the adjusted Bonferroni threshold highlighted in red (**a**) and associated QQ plot (**b**)

**Fig. 4:**
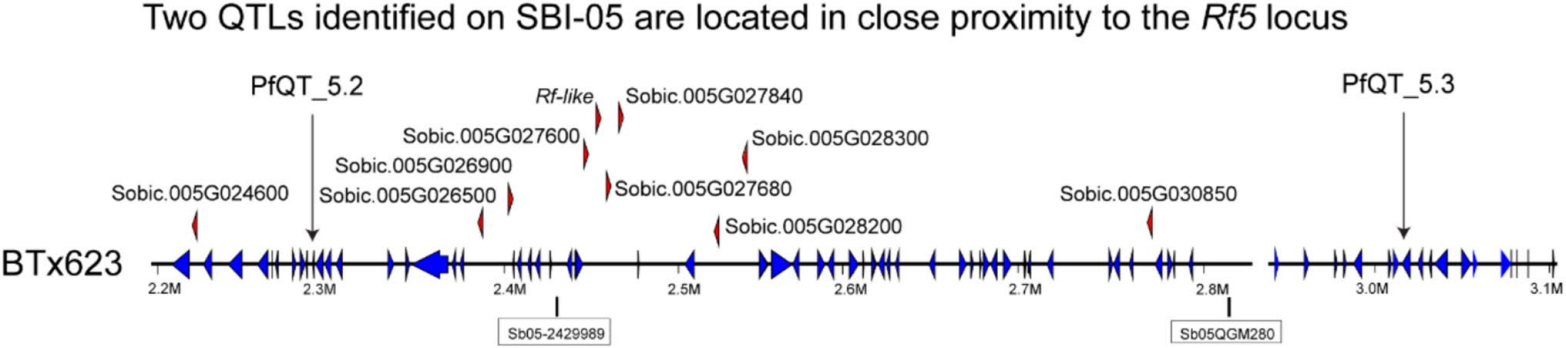
Co-localization of QTLs PfQT-5.2 and 5.3 with the *Rf5* locus on SBI-05

An association analysis was undertaken with BLINK (Huang et al. 2019) and detected 43 significant SNPs, above the adjusted Bonferroni significance threshold (Fig. 3 and Table 2). The significant SNPs were distributed across all chromosomes, with chromosomes 2 and 5 containing the highest number (7 and 8, respectively) and chromosome 7 the least (with only 1 significant SNP identified). The cM locations of the significant SNPs were predicted from the sorghum consensus genetic linkage map (Mace and Jordan 2011). Over 20% of the significant SNPs co-located within a 2 cM window with candidate genes identified from either previous studies on major fertility restoration genes in sorghum (*Rf*_*5*_ candidate gene on SBI-05) or genes under differential expression during anther formation identified in Dhaka et al. (2020) (see Table 2). Two QTLs, PfQT_5.2 and PfQT_5.3, were located within the RFL/PPR gene cluster on chromosome SBI-05 in close proximity to the region delimiting the *Rf5* locus (Fig. 4).

**Table 2:** Summary of the partial fertility QTL identified and positional candidate genes relative to positions of mapped major restorer of fertility genes

QTL Name	Chr	bp	Predicted cM*	− LOG10P	Candidate genes within 2 cM
PfQT_1.1	1	9,834,036	25.7	7.05	*OsINV4 (Sobic.001G099700)*^ϯ^
PfQT_1.2	1	14,774,613	38.4	7.61	
PfQT_1.3	1	54,119,665	60.5	10.21	
PfQT_1.4	1	68,289,390	125.7	18.10	*ZEP1 (Sobic.001G415800)*^ϯ^
PfQT_2.1	2	55,667,693	81	11.55	
PfQT_2.2	2	56,293,045	83.8	7.02	
PfQT_2.3	2	60,652,519	123.1	6.56	
PfQT_2.4	2	63,508,163	141	11.78	
PfQT_2.5	2	68,226,076	155.2	7.47	
PfQT_2.6	2	71,527,180	176.1	23.27	*OIP30 (Sobic.002G353500)*^ϯ^
PfQT_2.7	2	75,422,068	192.5	10.38	
PfQT_3.1	3	52,261,094	60.4	6.19	*OsMST8 (Sobic.003G192200)*^ϯ^
PfQT_3.2	3	53,198,376	64.8	7.73	
PfQT_3.3	3	56,759,289	83.6	7.21	
PfQT_3.4	3	68,112,264	139.2	6.77	*MID1 (Sobic.003G365600)*^ϯ^, *OsNek5 (Sobic.003G366300)*^ϯ^
PfQT_3.5	3	70,705,744	151.6	8.51	
PfQT_4.1	4	1,779,839	14.2	8.61	
PfQT_4.2	4	2,801,430	20.5	7.53	
PfQT_4.3	4	10,204,655	62.8	6.99	*OsPCBP (Sobic.004G100900)*^ϯ^, *HEI10 (Sobic.004G101500)*^ϯ^
PfQT_4.4	4	57,829,750	106.9	7.73	
PfQT_4.5	4	60,854,468	110.6	23.52	
PfQT_5.1	5	453,977	3	11.53	
PfQT_5.2	5	2,301,604	17.3	10.28	*Rf5 (Sobic.005G027840)*
PfQT_5.3	5	3,084,145	22.7	8.47	
PfQT_5.4	5	63,658,054	74.1	6.64	
PfQT_5.5	5	67,028,867	93	5.40	
PfQT_5.6	5	67,573,378	69.5	6.96	
PfQT_5.7	5	68,964,625	97.6	9.10	
PfQT_5.8	5	71,712,867	118.5	6.16	
PfQT_6.1	6	2,144,465	24.3	31.52	
PfQT_6.2	6	51,899,891	104.4	9.12	*OsPLIM2b (Sobic.006G159000)*^ϯ^
PfQT_6.3	6	54,298,205	128.7	6.59	*OsCDPK7 (Sobic.006G192500)*^ϯ^, *HTH1 (Sobic.006G185600)*^ϯ^
PfQT_7.1	7	8,301,063	66.3	5.60	*OsZIP4 (Sobic.007G075700)*^ϯ^
PfQT_8.1	8	1,206,988	14.8	8.73	
PfQT_8.2	8	59,829,272	67.1	5.78	
PfQT_8.3	8	60,449,286	103.8	6.98	
PfQT_9.1	9	1,182,457	14.2	9.46	*RPA1c (Sobic.009G012600)*^ϯ^
PfQT_9.2	9	4,762,731	53.4	7.04	
PfQT_9.3	9	52,687,515	105.2	11.48	*OsMSH4 (Sobic.009G185700)*^ϯ^
PfQT_10.1	10	3,827,432	33.8	8.78	
PfQT_10.2	10	7,264,827	37.9	6.06	
PfQT_10.3	10	51,534,736	67.4	5.83	
PfQT_10.4	10	56,987,810	89.7	8.81	

*Based on the sorghum consensus genetic linkage map (Mace and Jordan 2011)

^ϯ^Based on the set of 63 candidate genes identified byDhaka et al. (2020)

The Rf-like PPR genes in this region are shown as red triangles, and their names are given above the triangles; other non-Rf-like genes are shown as blue triangles. The two markers delimiting the *Rf5* locus (Jordan et al. 2011) are indicated.

### Principal component analysis, relative diversity and linkage disequilibrium

**Fig. 5:**
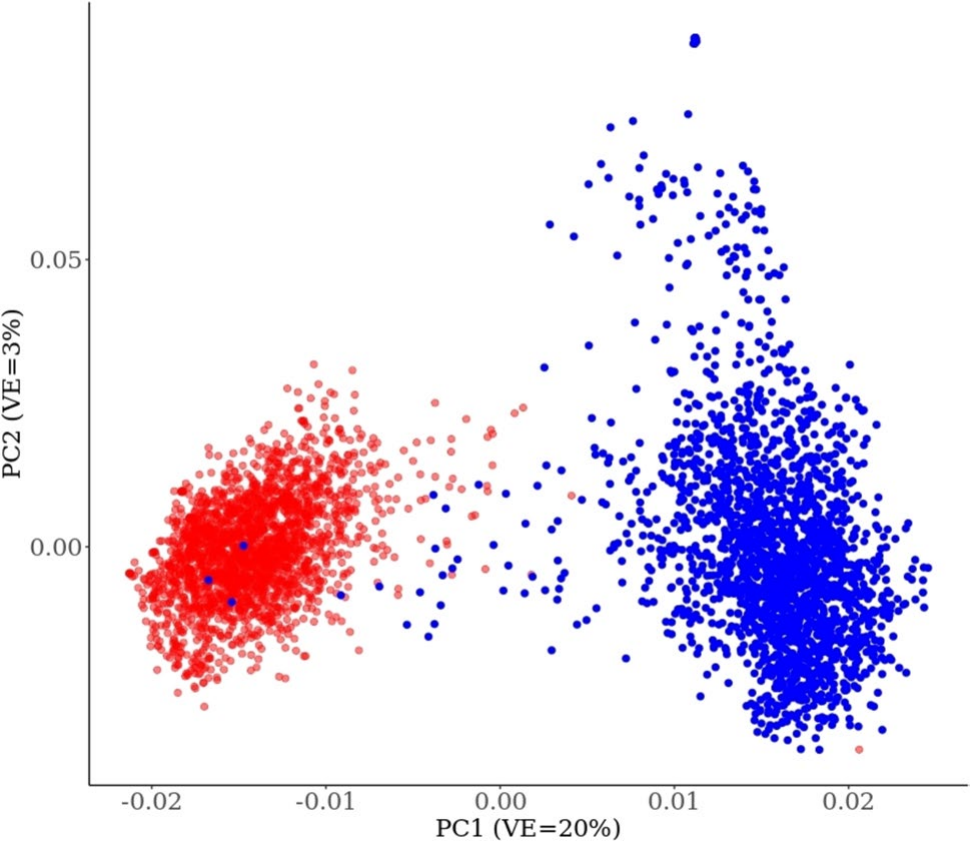
Principal component analysis of elite male and female inbreed lines from the UQ/DAF/GRDC sorghum breeding programme, specifically 2219 advanced *A*/*B* lines (in red) and 2135 *R* lines (in blue) (colour figure online)

Figure 5 shows the principal component analysis for germplasm set 2. The female and male parents clearly cluster into two separate groups on the first principal component, which explains ~ 20% of the variance.

Table 3 shows the nucleotide diversity (*π*) within the *A*/*B* and *R* line pools from the UQ/DAF/GRDC breeding programme. Average diversity within the female parent pool at 0.10 was ~ 23% lower than that of the male parent pool (0.12). Although relative diversity varied between chromosomes, in all but one chromosome (where the diversity values were the same) the *R* lines had higher diversity. The diversity of the *A*/*B* lines was not reduced relative to *R* lines in linkage groups that contained fertility restoration genes.

**Table 3:** Nucleotide diversity (*π*) of elite *A*/*B* and *R* lines from the UQ/DAF/GRDC breeding programme with the presence of *Rf* genes indicated

Chromosome	*π R*	*π B*	*π* % difference	*Rf* genes on LG
1	0.12	0.07	38	
2	0.13	0.11	16	*Rf2*
3	0.15	0.11	26	
4	0.12	0.10	16	*Pf*_1_*
5	0.12	0.10	15	*Rf5*
6	0.14	0.10	31	
7	0.12	0.07	35	
8	0.12	0.12	0	*Rf1*
9	0.12	0.07	37	
10	0.11	0.10	11	
Average	0.12	0.10	23	

*Pf_*1*_ is a partial fertility gene mapped byJordan et al (2011)

**Fig. 6:**
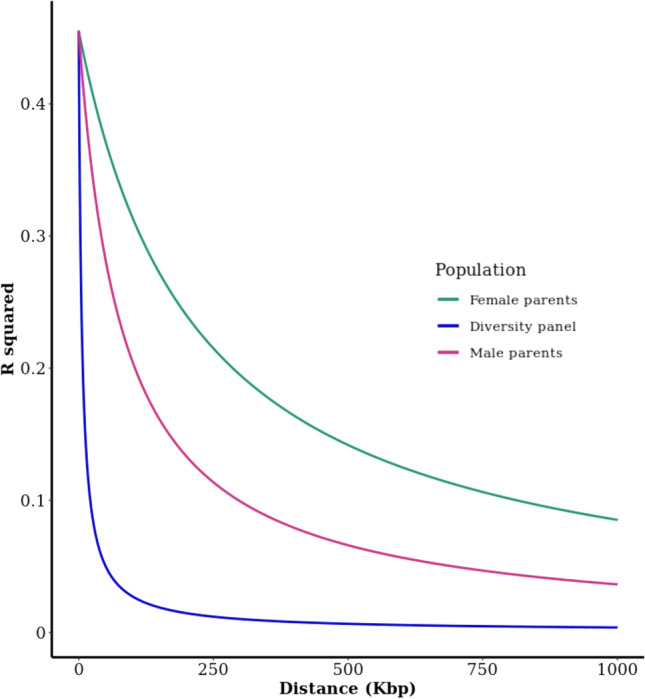
LD decay determined by squared correlations of allele frequencies (*r*.^2^) against distance between polymorphic sites in *A*/*B* (Female), *R* (Male) and a diversity panel (described previously in Tao et al. 2020)

Figure 6 shows a plot of LD decay determined by squared correlations of allele frequencies (*R*^2^) against distance between polymorphic sites in the elite *A*/*B* and *R* lines from the breeding programme contrasted with the Australian sorghum diversity panel (Tao et al. 2020). The LD decays rapidly in the diversity panel, dropping to *R*^2^ < 0.1 at ~ 20 kb, whereas LD did not decay to *R*^2^ < 0.1 until ~ 200 kb in the male parental pool, and > 600 kb in the female parental pool. Specifically, at the distance of 250 kb (which corresponds to a genetic distance of ~ 1 cM in the euchromatin (Mace et al. 2012), LD in the diversity panel is close to zero, whereas it is 0.09 in the elite *R* lines and almost double that Fig. (0.18) in the *A*/*B* lines.

## Discussion

In this paper we explored the previously undocumented genetic architecture of partial fertility in cytoplasmic male sterile lines of sorghum. High temperatures around flowering can induce partial fertility in cytoplasmic male sterile lines. Partial fertility in a commercial seed field causes a major problem for commercial seed companies potentially rendering hybrid seed unsaleable. While the genetic control of fertility restoration is well known and controlled by a relatively small number of major genes (Jordan et al. 2010, 2011), the control of partial fertility is poorly understood. In this study, we used GWAS on a panel of 2049 sorghum lines grown in six environments to identify 43 QTL for partial fertility and explore the genetic networks underlying the trait and the likely role of the trait in constraining genetic gain in commercial breeding programmes.

### Partial fertility is controlled by many minor genes and major restoration genes do not make a major contribution to the trait

A set of 2049 cytoplasmic male sterile lines were grown in six field trials at Emerald QLD such that flowering corresponded with the hottest part of the year, with temperatures with average maximum and average minimum temperatures around the flowering period were 35.4 °C and 21 °C, respectively. Freshly flowered segments of heads were rated on 36 separate days generating ~ 20,000 plot × day observations. Considerable variation in partial fertility was observed among CMS lines. However, pairwise correlations of sterility scores across days were high as was the broad sense heritability of the trait across trials (0.8). GWAS conducted on an across site BLUEs of sterility identified 43 significant marker trait associations indicating that partial fertility is a quantitative trait of moderate complexity. One of the potential explanations of partial fertility is that it is caused by subfunctional *Rf*-like PPR genes. Our evidence indicates this is not likely to be the case and that the situation is more complex. The number of QTL detected for this trait (43) is much larger than the number of known fertility restoration genes and only one of these four major fertility restoration genes, *Rf*_*5*_ (Jordan et al. 2011), located within 2 cM of a GWAS hit. Furthermore, the regions surrounding the GWAS hits were not found to be enriched for *Rf-like* PPR genes as would be expected if partial fertility were the result of subfunctional *Rf* genes.

### Candidate gene analysis suggests a range of gene networks are important in partial fertility

Dhaka et al. (2020) conducted an expression analysis study of different stages of sorghum anthers and combined this with information from rice to identify a list of candidates for engineering male fertility in sorghum. Given that partial fertility occurs in response to environmental conditions at flowering, it seems likely that some genes identified as being differentially expressed in anthers and associated with fertility in rice will be associated with the partial fertility phenotype. A comparison of the location of the significant SNPs from GWAS with the location of the genes identified in Dhaka et al. (2020) identified ~ 25% overlap, with 15 out of the 63 genes co-locating within a 2 cM window of the significant SNPs from this study (Table 2). The function of these genes may provide some indications of the gene networks involved in partial fertility.

One of the genes identified by Dhaka et al. (2020) was *Sobic.006G159000*, an orthologue of rice *OsPLIM2b*, which is directly implicated in cytoplasmic male sterility in rice. In rice, OsPLIM2b was shown to interact with the cytoplasmic male sterility-related protein kinase, OsNek3, and the transcripts of both genes were found to be preferentially expressed in anthers in bi- to tri-cellular pollen (Fujii et al. 2009). Although *OsNek3* was not close to a significant SNP from GWAS in this study, the closely related *OsNEK5* was less than 1 cM from a significant SNP.

Three candidate genes for sterility, *Sobic.003G192200* (rice *OsMST8*), *Sobic.001G099700* (rice *OsINV4*) and *Sobic.004G100900* (rice *OsPCBP*) implicated in carbohydrate metabolism in the anthers, fall within 2 cM of significant SNPs from GWAS. In rice, *OsINV4*, an anther-specific cell wall acid invertase gene, and *OsMST8*, an anther-specific monosaccharide transporter, are downregulated by cold, resulting in pollen sterility due to interference in starch storage (Mamun et al. 2006). In addition, *OsPCBP* is a pollen expressed gene in rice that encodes a calmodulin-binding protein involved in calcium signalling and localized to amyloplasts (Zhang et al. 2012). Transformation experiments indicate that disruption of this gene causes failure of pollen development, likely through disruption of starch accumulation (Zhang et al. 2012).

A further four candidate genes for sterility, based on function in rice and Arabidopsis, *Sobic.001G415800* (rice *ZEP1*), *Sobic.004G101500* (rice *HEI10*), *Sobic.002G353500* (rice *OIP30*) and *Sobic.009G012600* (rice *RPA1c*), are implicated in meiosis and DNA replication. In rice, ZEP1 is critical for controlling crossovers during meiosis (Wang et al. 2010). Its function is closely linked to that of HEI10 whose immunolocalization signals always overlap with ZEP1 signals (Wang et al. 2012). *RPA1c* is involved in regulating crossover formation and DNA repair in rice. It is one of the subunits of Replication protein A (RPA), a heterotrimeric protein complex that binds single-stranded DNA. In plants, multiple genes encode the three RPA subunits (*RPA1*, *RPA2* and *RPA3*), and in combination with the partially sterile *RPA1a*, *RPA1c* has been demonstrated to result in sterility in Arabidopsis (Aklilu et al. 2013). Finally, OIP30 is a helicase A class of enzyme that may be a potential substrate for the pollen predominant OsCPK25/26 in rice (Wang et al. 2011).

A further two candidate genes from the gene set identified by Dhaka et al. (2020) that fell within 2 cM of a significant hit from GWAS in the current study were related to other physiological functions in the rice anthers. *Sobic.003G365600* (rice *MID1*/*OsARM1*) is a transcriptional regulator that promotes rice male development under drought by modulating the expressions of drought-related and anther developmental genes (Wang et al. 2017). *Sobic.006G185600* (rice *HTH1*) is highly expressed in the epidermis of the anther in rice where it is involved in anther cutin biosynthesis and is required for pollen fertility in rice. Its reduced expression results in abortion due to a collapsed anther wall (Xu et al. 2017).

### Partial fertility, rather than the frequency of restorer genes, imposes constraints on the genetic diversity of female parents in hybrid breeding programmes

The genetic diversity of the female, or *A*/*B* line, populations of hybrid breeding programmes are low relative to the male or *R* line parents (Crozier et al. 2020) as demonstrated by the breeding populations in this study. Linkage disequilibrium (LD) decays much more slowly in female parents than male parents from germplasm set 2, and as expected both decay more slowly than in a sample of global sorghum diversity (Tao et al. 2020). In the diversity set *R*^2^ declines to zero at ~ 250 kb, while at the same distance *R*^2^ in the female population is ~ 0.2, double that of the male population (~ 0.1). The extent of LD in a population is the result of the complex interplay of factors such as selection, admixture, linkage and genetic drift. Typically, populations with small effective population size (Ne) experience more genetic drift than larger populations with closely linked loci indicating population sizes over the historical past, while loosely linked loci signify Ne in the immediate past (Hayes et al. 2003; Hill and Robertson 1966). The divergence in LD identified between the parental populations, at both close and loosely linked loci, suggests that Ne has been low in female, B, lines compared with male, R, lines in both the recent and historical past.

It is often stated that the major reason for the low genetic diversity within female parent lines is the fact that most sorghum landraces and germplasm lines are restorers of cytoplasmic male sterility (Menz et al. 2004). However, given that restoration of *A*_1_ cytoplasm is under the control of a small number of major genes (Jordan et al. 2010, 2011), it would seem to be relatively easy to remove these genes via phenotypic selection in test crosses or more recently by selection with molecular markers. This conjecture is further strengthened by the observation that the genetic diversity in *B* line material was not significantly lower than *R* lines for linkage groups containing restorer genes compared with linkage groups that did not. It seems unlikely therefore that frequency of restorer genes is sufficient to explain the observed differences in genetic diversity between the male and female parental pools. We propose that the difference in genetic diversity between the pools is primarily driven by the genetic architecture of partial fertility. The large number of loci influencing partial fertility identified in this study coupled with their environmental and dosage-dependent expression would make selection against partial fertility difficult. At the same time, the financial consequences of fertility breakdown are large, leading commercial breeders to be conservative in their crossing decisions when developing new female parent lines. This in turn has resulted in low diversity and high LD of female parent lines.

## Conclusions

In contrast to fertility restoration, temperature-induced partial fertility in *A*_1_ cytoplasm of sorghum is under complex multigenic control. It involves nuclear genes from a range of different networks influencing a variety of biological processes, which are distinct from the major restorer genes that are almost exclusively PPR genes that act via mediation of gene expression in mitochondria. A possible explanation for these results is that in female parent lines, high temperatures partially impair the expression or function of sterility-inducing genes present in the mitochondria exposing variation in the downstream networks of genes that influence pollen production.

The presence of at least 43 regions of the sorghum genome interacting with the environment to influence partial fertility combined with the large negative commercial impacts of fertility in female parents appears to be the major cause of lower genetic variation in female vs male parents. The low variation and higher LD in female parents are likely to have constrained genetic gain and made introgression of new traits difficult. In the future, it should be feasible to use genomic prediction to introduce new variation while maintaining sterility.

## Supplementary Information

Below is the link to the electronic supplementary material.

Supplementary file 1 (DOCX 17 KB)

## Data Availability

Data are available upon request.

## References

[CR1] Ahmadikhah A, Karlov GI (2006) Molecular mapping of the fertility-restoration gene *Rf4* for WA-cytoplasmic male sterility in rice. Plant Breed 125:363–367. 10.1111/j.1439-0523.2006.01246.x

[CR2] Aklilu BB, Soderquist RS, Culligan KM (2013) Genetic analysis of the Replication Protein A large subunit family in Arabidopsis reveals unique and overlapping roles in DNA repair, meiosis and DNA replication. Nucleic Acids Res 42:3104–3118. 10.1093/nar/gkt1292 24335281 10.1093/nar/gkt1292 PMC3950690

[CR3] Barkan A, Small I (2014) Pentatricopeptide repeat proteins in plants. Annu Rev Plant Biol. 65:415–442. 10.1146/annurev-arplant-050213-040159 24471833 10.1146/annurev-arplant-050213-040159

[CR4] Batch JJ, Morgan DG (1974) Male-sterility induced in barley by photoperiod. Nature 250:165–167. 10.1038/250165a0

[CR5] Bohra A, Jha UC, Adhimoolam P, Bisht D, Singh NP (2016) Cytoplasmic male sterility (CMS) in hybrid breeding in field crops. Plant Cell Rep 35:967–993. 10.1007/s00299-016-1949-3 26905724 10.1007/s00299-016-1949-3

[CR6] Brooking I (1976) Male sterility in *Sorghum bicolor* (L.) Moench induced by low night temperature. I. Timing of the stage of sensitivity. Funct Plant Biol 3:589–596. 10.1071/PP9760589

[CR7] Browning BL, Browning SR (2016) Genotype imputation with millions of reference samples. Am J Hum Genet 98:116–126. 10.1016/j.ajhg.2015.11.020 26748515 10.1016/j.ajhg.2015.11.020 PMC4716681

[CR8] Browning BL, Zhou Y, Browning SR (2018) A one-penny imputed genome from next-generation reference panels. Am J Hum Genet 103:338–348. 10.1016/j.ajhg.2018.07.015 30100085 10.1016/j.ajhg.2018.07.015 PMC6128308

[CR9] Butler D, Cullis B, Gilmour A, Gogel B (2009) Asreml. R package version 3

[CR10] Chase CD (2007) Cytoplasmic male sterility: a window to the world of plant mitochondrial-nuclear interactions. Trends Genet 23:81–90. 10.1016/j.tig.2006.12.004 17188396 10.1016/j.tig.2006.12.004

[CR11] Crozier D, Hoffmann L, Klein PE, Klein RR, Rooney WL (2020) Predicting heterosis in grain sorghum hybrids using sequence-based genetic similarity estimates. J Crop Improv 34:600–617. 10.1080/15427528.2020.1748152

[CR12] Cui XQ, Wise RP, Schnable PS (1996) The *rf2* nuclear restorer gene of male-sterile T-cytoplasm maize. Science 272:1334–1336. 10.1126/science.272.5266.1334 8650543 10.1126/science.272.5266.1334

[CR13] Cullis BR, Smith AB, Coombes NE (2006) On the design of early generation variety trials with correlated data. J Agric Biol Environ Stat 11:381–393. 10.1198/108571106X154443

[CR15] Danecek P, Auton A, Abecasis G, Albers CA, Banks E, Depristo MA, Handsaker RE, Lunter G, Marth GT, Sherry ST, Mcvean G, Durbin R (2011) The variant call format and VCFtools. Bioinformatics 27:2156–2158. 10.1093/bioinformatics/btr330 21653522 10.1093/bioinformatics/btr330 PMC3137218

[CR16] Dhaka N, Krishnan K, Kandpal M, Vashisht I, Pal M, Sharma MK, Sharma R (2020) Transcriptional trajectories of anther development provide candidates for engineering male fertility in sorghum. Sci Rep-Uk. 10.1038/s41598-020-57717-0 10.1038/s41598-020-57717-0 PMC6972786 31964983

[CR17] Doyle JJ, Doyle JL (1987) A rapid DNA isolation procedure for small quantities of fresh leaf tissue. Phytochem Bull 19:11–15

[CR18] Fishman L, Sweigart AL (2018) When two rights make a wrong: the evolutionary genetics of plant hybrid incompatibilities. Annu Rev Plant Biol 69:707–731. 10.1146/annurev-arplant-042817-040113 29505737 10.1146/annurev-arplant-042817-040113

[CR19] Fujii S, Yamada M, Toriyama K (2009) Cytoplasmic male sterility-related protein kinase, OsNek3, is regulated downstream of mitochondrial protein phosphatase 2C, DCW11. Plant Cell Physiol 50:828–837. 10.1093/pcp/pcp026 19224952 10.1093/pcp/pcp026

[CR20] Gaborieau L, Brown GG, Mireau H (2016) The propensity of pentatricopeptide repeat genes to evolve into restorers of cytoplasmic male sterility. Front Plant Sci. 10.3389/fpls.2016.01816 27999582 10.3389/fpls.2016.01816 PMC5138203

[CR21] Geddy R, Brown GG (2007) Genes encoding pentatricopeptide repeat (PPR) proteins are not conserved in location in plant genomes and may be subject to diversifying selection. BMC Genomics 8:130. 10.1186/1471-2164-8-130 17521445 10.1186/1471-2164-8-130 PMC1892557

[CR22] Hanson MR, Bentolila S (2004) Interactions of mitochondrial and nuclear genes that affect male gametophyte development. Plant Cell 16:S154–S169. 10.1105/tpc.015966 15131248 10.1105/tpc.015966 PMC2643387

[CR23] Hayes BJ, Visscher PM, McPartlan HC, Goddard ME (2003) Novel multilocus measure of linkage disequilibrium to estimate past effective population size. Genome Res 13:635–643. 10.1101/gr.387103 12654718 10.1101/gr.387103 PMC430161

[CR24] Hill WG, Robertson A (1966) The effect of linkage on limits to artificial selection. Genet Res 8:269–294. 10.1017/S0016672300010156 5980116

[CR25] Huang M, Liu XL, Zhou Y, Summers RM, Zhang ZW (2019) BLINK: a package for the next level of genome-wide association studies with both individuals and markers in the millions. Gigascience. 10.1093/gigascience/giy154 30535326 10.1093/gigascience/giy154 PMC6365300

[CR26] Itabashi E, Iwata N, Fujii S, Kazama T, Toriyama K (2011) The fertility restorer gene, *Rf2*, for Lead Rice-type cytoplasmic male sterility of rice encodes a mitochondrial glycine-rich protein. Plant J 65:359–367. 10.1111/j.1365-313x.2010.04427.x 21265890 10.1111/j.1365-313X.2010.04427.x

[CR27] Jordan DR, Mace ES, Henzell RG, Klein PE, Klein RR (2010) Molecular mapping and candidate gene identification of the Rf2 gene for pollen fertility restoration in sorghum [*Sorghum bicolor* (L.) Moench]. Theor Appl Genet 120:1279–1287. 10.1007/s00122-009-1255-3 20091293 10.1007/s00122-009-1255-3

[CR28] Jordan DR, Klein RR, Sakrewski KG, Henzell RG, Klein PE, Mace ES (2011) Mapping and characterization of *Rf*_*5*_: a new gene conditioning pollen fertility restoration in A_1_ and A_2_ cytoplasm in sorghum (*Sorghum bicolor* (L.) Moench). Theor Appl Genet 123:383–396. 10.1007/s00122-011-1591-y 21487690 10.1007/s00122-011-1591-y

[CR29] Kazama T, Okuno M, Watari Y, Yanase S, Koizuka C, Tsuruta Y, Sugaya H, Toyoda A, Itoh T, Tsutsumi N, Toriyama K, Koizuka N, Arimura S (2019) Curing cytoplasmic male sterility via TALEN-mediated mitochondrial genome editing. Nat Plants 5:722–730. 10.1038/s41477-019-0459-z 31285556 10.1038/s41477-019-0459-z

[CR30] Kim YJ, Zhang D (2018) Molecular control of male fertility for crop hybrid breeding. Trends Plant Sci 23(1):53–65. 10.1016/j.tplants.2017.10.001 29126789 10.1016/j.tplants.2017.10.001

[CR31] Kiyosawa A, Yonemaru JI, Mizuno H, Kanamori H, Wu J, Kawahigashi H, Goto K (2022) Fine mapping of *Rf5* region for a sorghum fertility restorer gene and microsynteny analysis across grass species. Breed Sci. 72(2):141–149. 10.1270/jsbbs.21057 36275935 10.1270/jsbbs.21057 PMC9522528

[CR32] Klein RR, Klein PE, Chhabra AK, Dong J, Pammi S, Childs KL, Mullet JE, Rooney WL, Schertz KF (2001) Molecular mapping of the *rf1* gene for pollen fertility restoration in sorghum (*Sorghum bicolor* L.). Theor Appl Genet 102:1206–1212. 10.1007/s001220100575

[CR33] Komori T, Ohta S, Murai N, Takakura Y, Kuraya Y, Suzuki S, Hiei Y, Imaseki H, Nitta N (2004) Map-based cloning of a fertility restorer gene, *Rf-1*, in rice (*Oryza sativa* L.). Plant J 37:315–325. 10.1046/j.1365-313x.2003.01961.x 14731253 10.1046/j.1365-313x.2003.01961.x

[CR34] Li MX, Yeung JMY, Cherny SS, Sham PC (2012) Evaluating the effective numbers of independent tests and significant p-value thresholds in commercial genotyping arrays and public imputation reference datasets. Hum Genet 131:747–756. 10.1007/s00439-011-1118-2 22143225 10.1007/s00439-011-1118-2 PMC3325408

[CR35] Luo DP, Xu H, Liu ZL, Guo JX, Li HY, Chen LT, Fang C, Zhang QY, Bai M, Yao N, Wu H, Wu H, Ji CH, Zheng HQ, Chen YL, Ye S, Li XY, Zhao XC, Li RQ, Liu YG (2013) A detrimental mitochondrial-nuclear interaction causes cytoplasmic male sterility in rice. Nat Genet 45:573-U157. 10.1038/ng.2570 23502780 10.1038/ng.2570

[CR36] Mace ES, Jordan DR (2011) Integrating sorghum whole genome sequence information with a compendium of sorghum QTL studies reveals uneven distribution of QTL and of gene-rich regions with significant implications for crop improvement. Theor Appl Genet 123:169–191. 10.1007/s00122-011-1575-y 21484332 10.1007/s00122-011-1575-y

[CR37] Mace ES, Singh V, Van Oosterom EJ, Hammer GL, Hunt CH, Jordan DR (2012) QTL for nodal root angle in sorghum (*Sorghum bicolor* L. Moench) co-locate with QTL for traits associated with drought adaptation. Theor Appl Genet 124:97–109. 10.1007/s00122-011-1690-9 21938475 10.1007/s00122-011-1690-9

[CR38] Madugula P, Uttam AG, Tonapi VA, Ragimasalawada M (2018) Fine mapping of Rf2, a major locus controlling pollen fertility restoration in sorghum A1 cytoplasm, encodes a PPR gene and its validation through expression analysis. Plant Breed 137:148–161. 10.1111/pbr.12569

[CR39] Mamun EA, Alfred S, Cantrill LC, Overall RL, Sutton BG (2006) Effects of chilling on male gametophyte development in rice. Cell Biol Int 30:583–591. 10.1016/j.cellbi.2006.03.004 16730464 10.1016/j.cellbi.2006.03.004

[CR40] Maunder A, Pickett R (1959) The genetic inheritance of cytoplasmic-genetic male sterility in grain sorghum. Agron J 51:47–49. 10.2134/agronj1959.00021962005100010016x

[CR41] McCormick RF, Truong SK, Sreedasyam A, Jenkins J, Shu SQ, Sims D, Kennedy M, Amirebrahimi M, Weers BD, McKinley B, Mattison A, Morishige DT, Grimwood J, Schmutz J, Mullet JE (2018) The *Sorghum bicolor* reference genome: improved assembly, gene annotations, a transcriptome atlas, and signatures of genome organization. Plant J 93:338–354. 10.1111/tpj.13781 29161754 10.1111/tpj.13781

[CR42] Melonek J, Stone J, Small I (2016) Evolutionary plasticity of restorer-of-fertility-like proteins in rice. Sci Rep 6:35152. 10.1038/srep35152 27775031 10.1038/srep35152 PMC5075784

[CR43] Melonek J, Zhou R, Bayer PE, Edwards D, Stein N, Small I (2019) High intraspecific diversity of restorer-of-fertility-like genes in barley. Plant J 97(2):281–295. 10.1111/tpj.14115 30276910 10.1111/tpj.14115 PMC7380019

[CR44] Menz MA, Klein RR, Unruh NC, Rooney WL, Klein PE, Mullet JE (2004) Genetic diversity of public inbreds of sorghum determined by mapped AFLP and SSR markers. Crop Sci 44:1236–1244. 10.2135/cropsci2004.1236

[CR45] Miller D, Pickett R (1964) Inheritance of partial male-fertility in *Sorghum vulgare* Pers. Crop Sci 4:1–4. 10.2135/cropsci1964.0011183X000400010001x

[CR46] Praveen M, Uttam GA, Suneetha N, Umakanth A, Patil JV, Madhusudhana R (2015) Inheritance and molecular mapping of *Rf*_*6*_ locus with pollen fertility restoration ability on A_1_ and A_2_ cytoplasms in sorghum. Plant Sci 238:73–80. 10.1016/j.plantsci.2015.05.020 26259176 10.1016/j.plantsci.2015.05.020

[CR47] Praveen M, Uttam AG, Tonapi VA, Ragimasalawada M (2018) Fine mapping of Rf2, a major locus controlling pollen fertility restoration in sorghum A(1) cytoplasm, encodes a PPR gene and its validation through expression analysis. Plant Breeding 137:148–161. 10.1111/pbr.12569

[CR48] Stephens JC, Holland RF (1954) Cytoplasmic male-sterility for hybrid sorghum seed production. Agron J 46:20–23. 10.2134/agronj1954.00021962004600010006x

[CR49] Tao YF, Zhao XR, Wang XM, Hathorn A, Hunt C, Cruickshank AW, van Oosterom EJ, Godwin ID, Mace ES, Jordan DR (2020) Large-scale GWAS in sorghum reveals common genetic control of grain size among cereals. Plant Biotechnol J 18:1093–1105. 10.1111/pbi.13284 31659829 10.1111/pbi.13284 PMC7061873

[CR50] Walkowiak S, Gao L, Monat C et al (2020) Multiple wheat genomes reveal global variation in modern breeding. Nature 588:277–283. 10.1038/s41586-020-2961-x 33239791 10.1038/s41586-020-2961-x PMC7759465

[CR51] Wang M, Wang K, Tang D, Wei C, Li M, Shen Y, Chi Z, Gu M, Cheng Z (2010) The central element protein ZEP1 of the synaptonemal complex regulates the number of crossovers during meiosis in rice. Plant Cell 22:417–430. 10.1105/tpc.109.070789 20154151 10.1105/tpc.109.070789 PMC2845403

[CR52] Wang CW, Chen WC, Lin LJ, Lee CT, Tseng TH, Leu WM (2011) OIP30, a RuvB-like DNA helicase 2, is a potential substrate for the pollen-predominant OsCPK25/26 in rice. Plant Cell Physiol 52:1641–1656. 10.1093/pcp/pcr094 21771866 10.1093/pcp/pcr094

[CR53] Wang K, Wang M, Tang D, Shen Y, Miao C, Hu Q, Lu T, Cheng Z (2012) The role of rice HEI10 in the formation of meiotic crossovers. PLoS Genet 8:e1002809. 10.1371/journal.pgen.1002809 22792078 10.1371/journal.pgen.1002809 PMC3390396

[CR54] Wang F-Z, Chen M-X, Yu L-J, Xie L-J, Yuan L-B, Qi H, Xiao M, Guo W, Chen Z, Yi K, Zhang J, Qiu R, Shu W, Xiao S, Chen Q-F (2017) OsARM1, an R2R3 MYB transcription factor, is involved in regulation of the response to arsenic stress in rice. Front Plant Sci. 10.3389/fpls.2017.01868 29163593 10.3389/fpls.2017.01868 PMC5670359

[CR55] Xu Y, Liu S, Liu Y, Ling S, Chen C, Yao J (2017) HOTHEAD-Like HTH1 is involved in anther cutin biosynthesis and is required for pollen fertility in rice. Plant Cell Physiol 58:1238–1248. 10.1093/pcp/pcx063 28838125 10.1093/pcp/pcx063

[CR56] Zhang Q, Li Z, Yang J, Li S, Yang D, Zhu Y (2012) A calmodulin-binding protein from rice is essential to pollen development. J Plant Biol 55:8–14. 10.1007/s12374-011-9184-5

[CR57] Zhang C, Dong S-S, Xu J-Y, He W-M, Yang T-L (2019) PopLDdecay: a fast and effective tool for linkage disequilibrium decay analysis based on variant call format files. Bioinformatics 35:1786–1788. 10.1093/bioinformatics/bty875 30321304 10.1093/bioinformatics/bty875

