# Supplementary material for: The genetic architecture of temperature-induced partial fertility restoration in A1 cytoplasm in sorghum (Sorghum bicolor (L.) Moench)
Source: Theor Appl Genet. 2025 Jul 2;138(7):170. doi: 10.1007/s00122-025-04946-4 (PMC12222369; doi:10.1007/s00122-025-04946-4)
Supplement: Supplementary file 1 — (DOCX 17 KB) [file 122_2025_4946_MOESM1_ESM.docx]

**Supplementary Information**

ESM Table S1: Genotype concurrence between different field trials across years

|  | Emer1403 | Emer1404 | Emer1505 | Emer1506 | Emer1607 | Emer1704 |
| --- | --- | --- | --- | --- | --- | --- |
| Emer1403 | 593 | 530 | 510 | 510 | 21 | 8 |
| Emer1404 | 530 | 603 | 517 | 518 | 20 | 7 |
| Emer1505 | 510 | 517 | 780 | 723 | 55 | 17 |
| Emer1506 | 510 | 518 | 723 | 783 | 57 | 17 |
| Emer1607 | 21 | 20 | 55 | 57 | 803 | 20 |
| Emer1704 | 8 | 7 | 17 | 17 | 20 | 682 |
